# Exploring the relationship between malnutrition and the systemic immune-inflammation index in older inpatients: a study based on comprehensive geriatric assessment

**DOI:** 10.1186/s12877-023-04604-8

**Published:** 2024-01-04

**Authors:** Yu-Cen Ma, Yan-Min Ju, Meng-Yu Cao, Di Yang, Ke-Xin Zhang, Hong Liang, Ji-Yan Leng

**Affiliations:** Department of Cadre Ward, The First Hospital of Jilin University, Changchun, 130021 China

**Keywords:** Systemic immune-inflammatory index, Malnutrition, Comprehensive geriatric assessment, Mini-nutritional assessment

## Abstract

**Background:**

Malnutrition is a prevalent and major challenge among senior citizens, possibly due to the continual low-grade inflammatory state of the body. A novel inflammatory parameter, the systemic immune-inflammation index (SII), is highly valuable in evaluating and predicting the prognosis of a wide range of diseases. This study aims to explore the significance of the SII in assessing malnutrition in older inpatients.

**Methods:**

This retrospective study included 500 senior hospitalized patients who met the inclusion and exclusion criteria from the Comprehensive Geriatric Assessment database of the First Hospital of Jilin University. The Mini-Nutritional Assessment (MNA) questionnaire was used to evaluate the nutritional status of patients. The SII was calculated using complete blood counts, and we performed natural logarithm transformation of the SII [ln(SII)]. Multivariable logistic regression analysis was used to identify the association between ln(SII) and malnutrition. To ensure the stability of the findings, a sensitivity analysis was conducted.

**Results:**

The 500 patients had a mean age of 77.29 ± 9.85 years, and 68.6% were male. In accordance with the MNA, 30.4% of the patients were malnourished or at risk of malnutrition, and patients in this group had considerably greater levels of ln(SII) than patients with adequate nutrition (*P* < 0.001). The optimum ln(SII) cutoff value for patients with malnutrition or at risk of malnutrition was 6.46 (SII = 635.87) with 46.7% sensitivity and 80.2% specificity [95% CI: 0.613–0.721, AUC: 0.667, P < 0.001]. Multivariable logistic regression demonstrated that ln(SII) was an independent risk factor for the risk of malnutrition or malnutrition in older individuals (OR 3.984, 95% CI: 2.426–6.543, *P* < 0.001). Other metrics from the geriatric comprehensive assessment, including body mass index, calf circumference, fat ratio, activities of daily living and instrumental activities of daily living, and geriatric depression scale scores, were also independently correlated with nutritional status.

**Conclusions:**

According to our research, a high SII is an independent predictor of older inpatient malnutrition, and the SII aids in screening for malnutrition and may be a potential target for intervention. Comprehensive geriatric assessment parameters such as BMI, calf circumference, fat ratio, activities of daily living and depression were also linked to malnutrition.

## Background

Malnutrition (undernutrition), an imbalanced nutritional status resulting from insufficient intake of nutrients to meet normal physiological requirement, is one of the most prevalent issues in the current aging society. According to a survey, the prevalence of malnutrition among older people in the community is 3.10%, whereas it is 22.00% among hospitalized older patients [1]. Older people are more likely to suffer from malnutrition as a result of chronic comorbidities, dental problems, mental health issues, etc., while malnutrition further exacerbates immune dysfunction, triggering a variety of adverse events, delaying recovery, increasing hospitalization duration, expenditures, and even mortality [2]. Therefore, it is necessary to access and intervene malnutrition in older inpatients as soon as possible after admission. Despite the complex etiology of disease-related malnutrition, inflammation has been found to be a key factor in its pathogenesis [3]. Chronic low-grade inflammation caused by tumors or other long-term illnesses in the older adults affects neural centers and appetite by causing striated muscle protein catabolism, preventing gastric emptying, and controlling hormones that are related to hunger, thus causing malnutrition [4, 5].

Inflammation across the body can be quantified by a variety of biochemical or hematological parameters, such as the number of inflammatory cells (neutrophils, lymphocytes, monocytes, etc.) alone or in the form of their ratio. Previous studies have accessed the ability of neutrophil-to-lymphocyte ratio (NLR) in predicting malnutrition in older people [6, 7]. The systemic immune-inflammation index (SII) is a novel comprehensive index based on the combination of NLR and platelet count. As a result, it is considered that SII may perform better as an inflammation biomarker in some illnesses than NLR [8]. It is often used as a predictor of malignancies [9], metabolic disorders [10] and cardio-cerebral events [11, 12]. However, the relationship between SII and malnutrition is currently unknown and we hypothesized that patients with malnutrition have higher SII.

In this background, we aimed to explore the significance of the SII in assessing the risk of malnutrition and malnutrition in older inpatients.

## Methods

### Study design

**Fig. 1:**
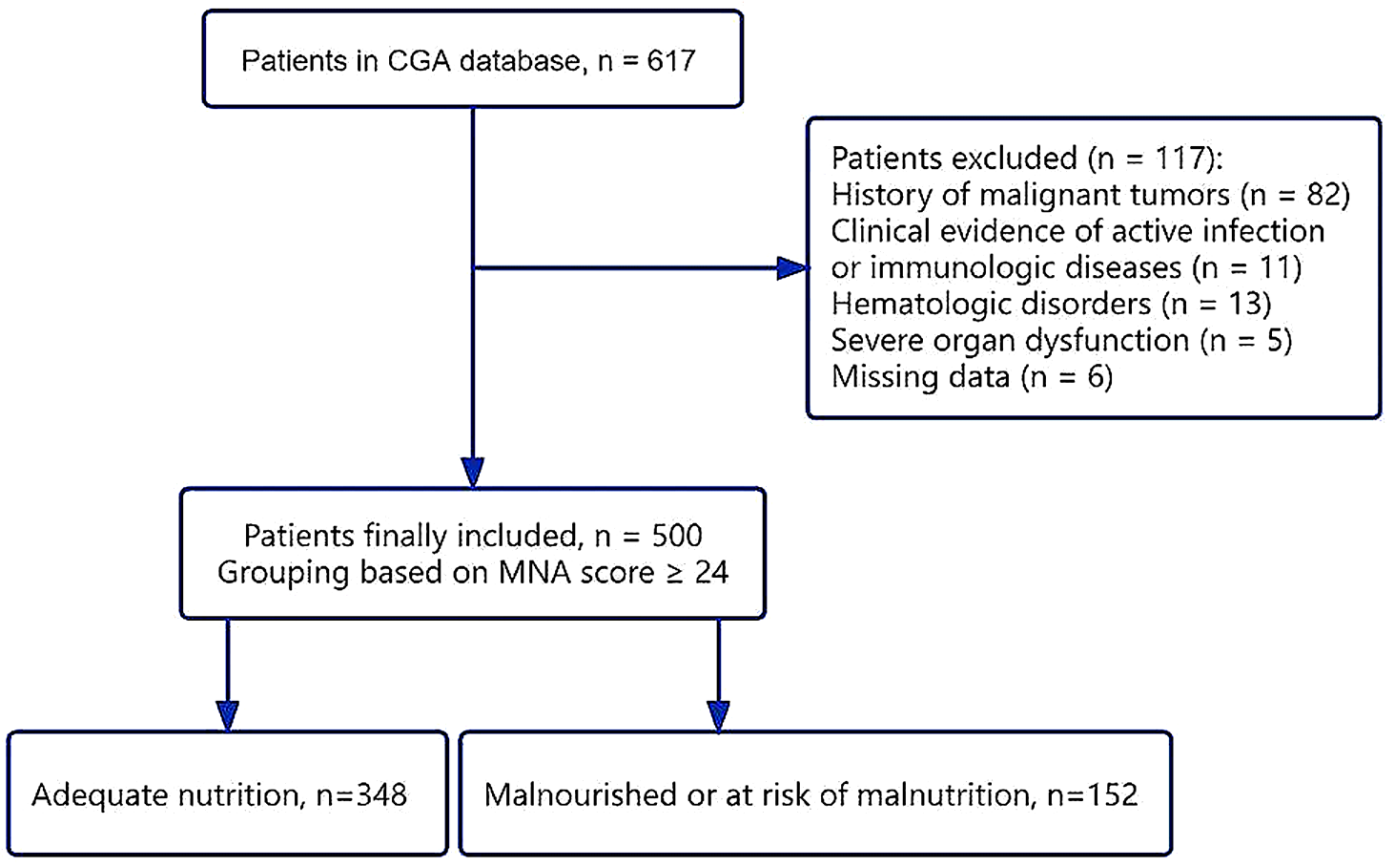
Flow chart showing the enrollment procedure for participants

We conducted a retrospective analysis using electronic medical records and the CGA database of patients admitted to the Cadre Ward Department of the First Hospital of Jilin University. The patients on the ward aged ≥ 60 years who completed CGA between December 2021 and October 2022 were included in the analysis. The exclusion criteria were as follows: with a history of malignant tumors; clinical evidence of active infection or immunologic diseases; hematologic disorders; severe organ dysfunction; or missing data. Ethical approval of this retrospective study was obtained from the Ethics Committee of the First Hospital of Jilin University. Figure 1 depicts a flow chart for patient selection.

### Comprehensive geriatric assessment

CGA is the core technology of geriatrics. Its main purpose is to thoroughly evaluate the general condition, comorbidity, polypharmacy, physical function, psychological status, cognitive function, and nutrition of patients with the help of a series of questionnaires, scales, and appropriate assessment tools (such as a stopwatch and hand-grip dynamometer). Taking into account the evaluation results and the patient’s needs to establish an individualized health management program for each patient, carrying out detailed and scientific guidance will help to prevent and control the development and progression of a variety of geriatric syndromes and diseases as well as enhance the quality of life for senior citizens. In our study, geriatricians with formal training in CGA performed the comprehensive assessments with informed consent from each patient or an appropriate agent.

### Data collection

The following information was collected from the CGA database: demographics (age and sex), smoking history, history of chronic diseases (hypertension, coronary heart disease, diabetes, stroke, and hyperuricemia diagnosed in the past or after admission), and anthropometric measurements [height, weight, body mass index (BMI), calf circumference (CC)]. BMI was calculated as weight (kilograms, kg) divided by height (meters, m) squared. CC was measured by placing an inelastic soft tapeline at the thickest region of the calf and measuring the circumference in a horizontal position (precise to 0.1 cm) while the patients were standing and their legs were separated by shoulder width. In addition, the concurrent use of five or more medications daily was defined as polypharmacy [13]. Body composition parameters such as fat ratio, fat mass, and fat-free mass were measured in the supine position of the individuals using an InBody S10 body composition analyzer (Biospace Co., Ltd., Seoul, Korea) based on bioelectrical impedance analysis (BIA). Activities of daily living and instrumental activities of daily living (ADLs & IADLs) assessed the ability to care for oneself physically and to use tools to perform activities of daily life, with 14 questions and a total score of 56. Higher scores indicated a more serious impairment of the subject’s capacity to carry out everyday tasks [14]. We screened for depression using the Geriatric Depression Scale (GDS), which has 30 items and a maximum score of 30, with a score higher than 10 suggesting depressive symptoms [15]. The Geriatric Depression Scale is reliable and valid, and it can be used to screen for depression in older Chinese adults including for those with mild cognitive impairment [16]. The Mini-Mental State Examination (MMSE) scale is one of the most commonly used cognitive function testing scales worldwide. For dementia screening, a score of 17 can be considered positive for those who are illiterate or uneducated, a score of 20 for those who have completed elementary school (with less than six years of education), and a score of 24 for those who have finished secondary school and above (with more than six years of education).

Laboratory tests such as CBC, albumin, and total cholesterol were obtained at admission from electronic medical records. After overnight fasting, the patients’ blood samples were taken the next morning. The SII was determined for all patients as platelet count × neutrophil count/lymphocyte count.

### Assessment of nutritional status

The nutritional status of patients was examined using the MNA scale, a nutritional assessment approach initially proposed and developed by Guigoz et al. in 1994. It has high sensitivity (97.9–100%) and specificity (69.5–100%) [17]. The application of MNA is encouraged by the European Society for Clinical Nutrition and Metabolism (ESPEN) for primary care physicians and health professionals caring for older people, particularly fragile individuals, at home, in nursing homes, or in hospitals. Additionally, it is often used as a nutritional assessment tool in Asian populations [18, 19]. The MNA scale consists of four parts with a total score of 30 points: anthropometry, self-reported health, dietary questionnaires, and clinical health. A patient who has an MNA score of less than 24 is either malnourished (MNA < 17) or at risk of malnutrition (MNA between 17 and 23.5) [20].

### Statistical analysis

IBM SPSS Statistics 25.0 was applied for data processing and statistical analysis. According to the MNA score, the participants were divided into two groups. Descriptive statistical analysis was performed on demographic information, smoking history, chronic diseases, number of drugs, laboratory tests, and CGA parameters. The Kolmogorov‒Smirnov test was used to determine the normality of continuous variables, and data that followed a normal distribution were reported as the mean standard deviation (mean ± SD), whereas those that did not were expressed as the median (interquartile range, IQR). Frequencies (percentages) were used to represent categorical variables. The difference in metrological data between the two groups was examined using the independent Student’s t test or the Mann‒Whitney U test, and categorical variables were compared using Pearson’s χ2 test. We performed natural logarithm transformation of the SII [ln(SII)]. The Spearman correlation coefficient was calculated to examine the relation between ln(SII) and nutritional parameters. The association between ln(SII) and nutrition was examined using both univariate and multivariate logistic regression analyses; in addition, a sensitivity analysis was carried out at the same time. Model 1 was unadjusted, and Model 2 was adjusted for age and history of coronary artery disease; Model 3 adjusted anthropometry and body composition based on Model 2; and Model 4 further adjusted the scale scores and polypharmacy based on Model 3. The area under the curve (AUC) value was calculated by drawing receiver operating characteristic (ROC) curve to identify the association between ln(SII) and malnutrition. The Youden index was used to determine the optimal cutoff value of ln(SII). All statistical analyses were performed on hypothesis testing, with α = 0.05 as the test level and *P* < 0.05 regarded as statistically significant.

## Results

Our study finally comprised a total of 500 subjects with 343 males (68.6%), 157 females (31.4%), and a mean age of 77.29 ± 9.85 years. Hypertension (62.4%), stroke (57.8%), and coronary heart disease (54.8%) were the most common chronic diseases. A total of 43.8% (n = 219) of the participants smoked. Polypharmacy was present in 29.8% (n = 149) of the individuals. The mean MNA score was 24.23 ± 3.03, and based on the MNA results, 348 (69.6%) subjects had an adequate nutritional status, and 152 (30.4%) subjects were at risk of malnutrition (n = 22, 4.4%) or malnourished (n = 130, 26%). According to the GDS scale, 19.6% (n = 98) of patients suffered from depression, whereas 17.4% (n = 87) had dementia as assessed by the MMSE.

Table 1 shows the demographics, clinical characteristics, and scale assessment of the subjects by nutritional status classification. Patients who were at risk of malnutrition or malnourished had a mean age of 82.14 ± 9.58 years, which was considerably older than patients who had an adequate nutritional status (*P* < 0.001). Patients with malnutrition or at risk of malnutrition were more likely to experience polypharmacy, have more serious impairment in activities of daily living, and suffer from cognitive decline and depressive symptoms than those with adequate nutrition. There were no statistically significant differences in sex or the prevalence of chronic comorbidities between the two groups.

**Table 1:** Demographics, clinical characteristics, and scale assessment of the subjects by nutritional status classification

Characteristics	Total	Adequate nutrition (MNA score 24–30) (n = 348)	Malnourished or at risk of malnutrition (MNA score ≤ 23.5) (n = 152)	*P*
**Age, years, mean ± SD**	77.29 ± 9.85	75.18 ± 9.20	82.14 ± 9.58	< 0.001
**Males, n (%)**	343(68.6%)	232(66.7%)	111(73.0%)	0.174
**Smoking, n (%)**	219(43.8%)	159(45.7%)	60(39.5%)	0.204
**Chronic diseases, n (%)**				
**Coronary heart disease**	274(54.8%)	180(51.7%)	94(61.8%)	0.040
**Hypertension**	312(62.4%)	214(61.5%)	98(64.5%)	0.548
**Diabetes**	158(31.6%)	115(33.0%)	43(28.3%)	0.298
**Stroke**	289(57.8%)	194(55.7%)	95(62.5%)	0.169
**Hyperuricemia**	137(27.4%)	95(27.3%)	42(27.6%)	1.000
**Polypharmacy, n (%)**	149(29.8%)	91(26.1%)	58(38.2%)	0.008
**ADLs & IADLs score, median (IQR)**	19.00(16.00–25.00)	17.00(14.00–22.00)	25.00(19.25-33.00)	< 0.001
**MNA score, mean ± SD**	24.23 ± 3.03	25.84 ± 1.24	20.54 ± 2.66	< 0.001
**MMSE score, mean ± SD**	25.06 ± 4.72	26.00 ± 3.89	22.92 ± 5.67	< 0.001
**Dementia, n (%)**	87(17.4%)	32(9.2%)	55(36.2%)	< 0.001
**GDS score, median (IQR)**	5.00(2.00–9.00)	4.00(1.00–7.00)	8.00(4.00–14.00)	< 0.001
**Depression, n (%)**	98(19.6%)	44(12.6%)	54(35.5%)	< 0.001

Abbreviations: ADLs & IADLs, Activities of daily living and instrumental activities of daily living; MNA, Mini-Nutritional Assessment; MMSE, Mini-Mental State Examination; GDS, Geriatric Depression Scale

The anthropometric and body composition parameters and laboratory findings of the individuals are shown in Table 2. Patients with malnutrition or at risk of malnutrition had substantially lower weight, BMI, CC, fat mass, and fat ratio than participants with normal nutritional status (all *P* < 0.001). The fat-free mass was also lower in the malnourished or at risk of malnutrition group, but the difference was not statistically significant. Furthermore, lymphocyte count, hemoglobin, albumin, and total cholesterol levels were considerably lower (all *P* < 0.001) in patients at risk of malnutrition or malnourished, whereas Ln(SII) was much higher (*P* < 0.001) than in the adequate nutrition group.

**Table 2:** The anthropometric and body composition parameters and laboratory findings of the individuals according to nutritional status

Parameters	Total	Adequate nutrition (MNA score 24–30)(n = 348)	Malnourished or at risk of malnutrition (MNA score ≤ 23.5) (n = 152)	*P*
**Height, m**	167.27 ± 7.40	167.29 ± 7.52	167.25 ± 7.13	0.957
**Weight, kg**	68.20 ± 11.05	70.21 ± 10.87	63.60 ± 10.09	< 0.001
**BMI, kg/m** ^**2**^	24.29 ± 3.07	24.99 ± 2.87	22.68 ± 2.91	< 0.001
**Fat mass, kg**	20.10 ± 6.46	21.58 ± 6.21	16.68 ± 5.69	< 0.001
**Fat-free mass, kg**	48.01 ± 8.00	48.44 ± 8.25	47.03 ± 7.32	0.072
**Fat ratio, %**	29.22 ± 6.95	30.60 ± 6.46	26.04 ± 7.02	< 0.001
**Calf circumference, cm**	35.67 ± 3.47	36.20 ± 3.01	34.81 ± 3.90	< 0.001
**WBC, 10^9/L**	6.20 ± 1.69	6.14 ± 1.64	6.35 ± 1.81	0.206
**Neutrophils, 10^9/L**	3.62(2.90–4.55)	3.54(2.81–4.45)	4.01(3.11–4.89)	0.001
**LC, 10^9/L**	1.60(1.21–2.01)	1.69(1.34–2.09)	1.29(0.98–1.77)	< 0.001
**Hemoglobin, g/L**	133.81 ± 19.20	137.31 ± 16.74	125.80 ± 21.93	< 0.001
**Platelet, 10^9/L**	191.50(158.00-236.00)	194.00(156.25-235.75)	187.50(158.25–237.00)	0.592
**Albumin, g/L**	37.78 ± 4.44	39.15 ± 3.60	34.66 ± 4.62	< 0.001
**TC, mg/dL**	4.41 ± 1.03	4.54 ± 1.02	4.10 ± 1.00	< 0.001
**Ln(SII)**	6.14 ± 0.60	6.01 ± 0.50	6.43 ± 0.69	< 0.001

Abbreviations: BMI, body mass index; CC, calf circumference; WBC, white blood cell; LC, lymphocyte count; TC, total cholesterol; ln (SII), the natural logarithmic form of systemic immune-inflammatory index

Table 3 displays the correlation analysis between ln(SII) and indicators of nutritional status. There was a negative correlation between ln(SII) and MNA scores (r = -0.331, *P* < 0.001). We also found negative correlations between ln(SII) and serum albumin (r = -0.214, *P* < 0.001), total cholesterol (r = -0.117, *P* = 0.009), fat mass (r = -0.139, *P* = 0.002), and fat ratio (r = -0.157, *P* < 0.001). Additionally, there was a connection between ln(SII) and the MMSE and GDS scores (r = -0.089, *P* = 0.047, and r = 0.101, *P* = 0.024, respectively).

**Table 3:** Correlation between ln(SII) and indicators of nutritional status

Variables	Ln (SII)	
	r	*P*
**Age, years**	0.068	0.127
**Weight, kg**	-0.007	0.884
**BMI, kg/m** ^**2**^	-0.094	0.035
**Calf circumference, cm**	-0.005	0.909
**Fat mass, kg**	-0.139	0.002
**Fat-free mass, kg**	0.020	0.660
**Fat ratio, %**	-0.157	< 0.001
**Hemoglobin, g/L**	-0.037	0.411
**Albumin, g/L**	-0.214	< 0.001
**TC, mg/dL**	-0.117	0.009
**MNA score**	-0.331	< 0.001
**ADLs & IADLs score**	0.080	0.075
**MMSE score**	-0.089	0.047
**GDS score**	0.101	0.024

Abbreviations: BMI, body mass index; CC, calf circumference; TC, total cholesterol; MNA, Mini-Nutritional Assessment; ADLs & IADLs, Activities of daily living and instrumental activities of daily living; MMSE, Mini-Mental State Examination; GDS, Geriatric Depression Scale

**Fig. 2:**
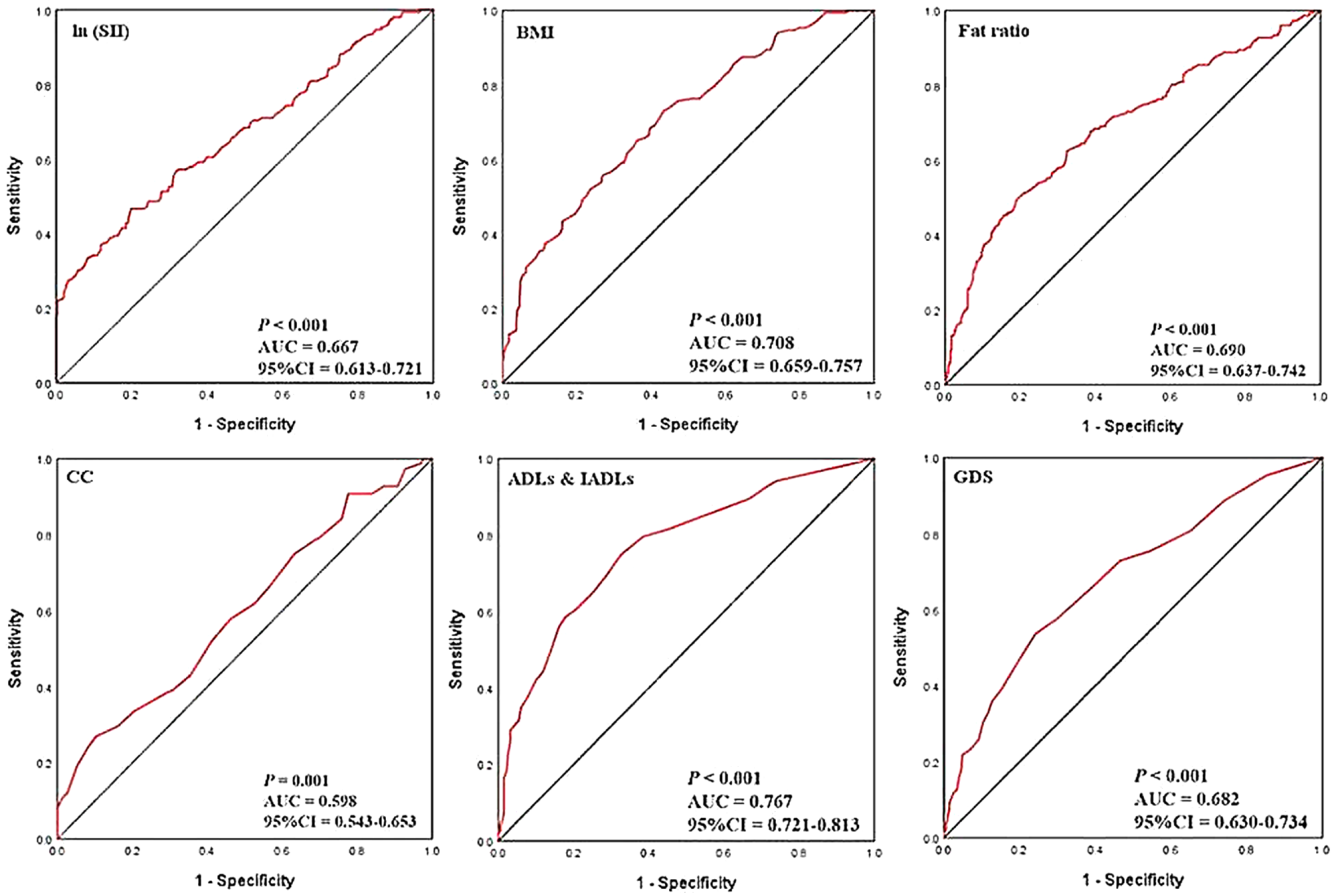
The receiver operating characteristic curves of ln(SII) and CGA indices for malnutrition or at risk of malnutrition

Univariable logistic regression analysis (Model 1) revealed that ln(SII) was an inflammatory marker highly linked with malnutrition or at risk of malnutrition, with an unadjusted OR of 3.630 (95% CI: 2.493–5.286, *P* < 0.001). Confounders with *P* < 0.1 in univariable logistic analysis were sequentially included in multivariable logistic models. Model 2 was adjusted for age and history of coronary heart disease, and ln(SII) was independently associated with malnutrition (OR 3.786; 95% CI: 2.538–5.649, *P* < 0.001). After adjusting for weight, BMI, CC, and fat ratio, the link between ln(SII) and malnutrition persisted in Model 3 (OR 3.831; 95% CI: 2.441–6.011, *P* < 0.001). On the basis of Model 3, Model 4 adjusted for ADL, MMSE, GDS scores, and polypharmacy. We observed that ln(SII) remained an independent risk factor for malnutrition (OR 3.984; 95% CI: 2.426–6.543, *P* < 0.001), and sensitivity analysis confirmed the stability of this conclusion (Table 4), indicating that for each unit increase in ln(SII) score, the risk of malnutrition was three times higher. Figure 2 depicts the ROC curves for variables with *P* < 0.05 in the results of multivariable logistic regression analysis Model 4. We found that the optimum ln(SII) cutoff value for patients with malnutrition or at risk of malnutrition was 6.46 (SII = 635.87) with 46.7% sensitivity and 80.2% specificity [95% CI: 0.613–0.721, AUC: 0.667, *P* < 0.001]. Aside from ln(SII), other CGA indices, such as BMI, CC, fat ratio, ADLs & IADLs, and GDS scores, were also independently associated with the risk of malnutrition or malnutrition.

**Table 4:** Univariable and multivariable logistic regression analysis of ln(SII) and malnutrition or at risk of malnutrition

	OR	95% confidence interval		*P*
		Lower bound	Upper bound	
**Model 1** ^**a**^	3.630	2.493	5.286	< 0.001
**Model 2** ^**b**^	3.786	2.538	5.649	< 0.001
**Model 3** ^**c**^	3.831	2.441	6.011	< 0.001
**Model 4** ^**d**^	3.984	2.426	6.543	< 0.001

^a^ unadjusted

^b^ adjusted for age and history of coronary heart disease

^c^ adjusted for b + weight, BMI, CC, and fat ratio

^d^ adjusted for c + ADLs & IADLs, MMSE, GDS scores, and polypharmacy

## Discussion

To the best of our knowledge, this is the first study to explore the significance of the SII in assessing the risk of malnutrition and malnutrition in older inpatients. We found patients with malnutrition have higher SII than those with adequate nutrition, and the risk of malnutrition increases significantly as SII above 635.87. After controlling for confounders, SII remained an independent risk factor for malnutrition. According to our findings, the SII aids in screening for malnutrition in older inpatients and may be a potential target for intervention.

The pathogenesis of malnutrition in older people is currently unknown. Researchers generally believe that aging first triggers a chronic inflammatory process [21], and persistent pro-inflammatory cytokines (including TNFα, CRP, IL-1β, and IL-6) increase the risk of inadequate nutrient intake by acting on neural center and endocrine system [5]. Since chronic immunological activation may have a larger demand for nutritional status, prolonged insufficient nutritional intake suppresses innate and adaptive immune system function [22], increasing vulnerability to illnesses and further aggravating malnutrition. In summary, immunological function, inflammation, and malnutrition are all interconnected and constitute a vicious cycle. In this regard, it becomes reasonable to assess malnutrition using systemic inflammatory biomarkers.

Research on SII and malnutrition is still in its early stages. Our study found low LC and high SII levels in people at risk of malnutrition or malnutrition based on the MNA questionnaire. After controlling for confounders, the SII remained an independent risk factor of malnutrition in older people. We tried to explain this association and noted that previous studies have reported an association between NLR and malnutrition [6, 7, 23, 24]. This may be due to the systemic inflammatory state usually characterized by an increase in neutrophils and monocytes (representing activation of inflammation) and a drop in lymphocytes (representing regulatory immunogenic responses) [23]. In addition, platelets are regarded as a non-specific inflammatory marker, and in chronic inflammatory states, cytokines such as IL-6 stimulate megakaryocytes to produce platelets, often leading to reactive thrombocytosis [25]. As mentioned above, the SII may become the gold standard for predicting malnutrition.

In our study, physical performance, psychological status and cognitive ability of older inpatients were all evaluated by means of CGA, and the indicators that may be related to nutritional status were emphatically analyzed. We discovered that, according to the MNA, 31.4% of older inpatients were malnourished or at risk of malnutrition, and nearly 20% of patients had depression or dementia. According to the results of the multivariable regression analysis, depression, activities of daily life, CC, and BMI were all independently associated with malnutrition. In earlier days, few studies have examined the relationship between individuals’ physical performance and psychological status while assessing malnutrition in hospitalized patients. Most concentrated on anthropometric measurements and nutritional screening scales [26, 27]. We also used novel technical means to compare the body composition of patients with different nutritional statuses and discovered that fat mass and fat ratio were significantly reduced in patients at risk of malnutrition or malnutrition. This finding is basically in line with Erdogan et al.’s conclusion [28] and is useful for early intervention and the detection of malnutrition.

Our study recommends the evaluation of SII and CGA as soon as possible after admission in older people, which can detect people at risk of malnutrition or malnutrition and perform early intervention. Nevertheless, the limitations of our study deserve consideration. First, this study was a retrospective analysis of data from a single center, and we did not follow up with patients. Second, although our CGA assessed the nutritional status of older adults in a more comprehensive way, nutritional indicators such as unintentional weight loss, triceps skinfold thickness, and waist circumference were not available from the database and were not included in this study. Furthermore, the link between high SII and malnutrition has not been fully explored, and future research might further investigate the relevant mechanisms by analyzing the dynamic changes in SII before and after nutritional intervention.

## Conclusion

According to our research, a high SII is an independent predictor of older inpatient malnutrition, and the SII aids in screening for malnutrition and may be a potential target for intervention. Geriatric Comprehensive Assessment parameters such as BMI, calf circumference, fat ratio, activities of daily living and depression were also linked to malnutrition.

## Data Availability

The datasets used and/or analysed during the current study are available from the corresponding author on reasonable request.

## Abbreviations

SII: Systemic immune-inflammation index
CGA: Comprehensive Geriatric Assessment
ESPEN: European Society for Clinical Nutrition and Metabolism
NLR: Neutrophil-to-lymphocyte ratio
CBC: Complete blood count
ADLs & IADLs: Activities of daily living and instrumental activities of daily living
MMSE: Mini-Mental State Examination
GDS: Geriatric Depression Scale
BMI: Body mass index
CC: Calf circumference
WBC: White blood cell
LC: Lymphocyte count
TC: Total cholesterol
BIA: Bioelectrical impedance analysis
